# Blockley Hospital

**Published:** 1859-01

**Authors:** 


BLOCKLEY HOSPITAL. — Notwithstanding the exertions of one of the former chief-resident physicians in this institution, and the numerous outcries of the press of the city, we are happy to say, that through the energy of Dr. Oliver, one of the Board of Guardians, clinical instruction has again been resumed, and facilities, unsurpassed by any hospital in this country, have been opened to students. The consulting corps appointed by the Board consists of the following gentlemen:—

*Physicians.*—Drs. Jos. Carson, J. B. Biddle, J. Aitken Meigs, J. Da Costa.

*Surgeons.*—Drs. J. Neill, D. H. Agnew, W. L. Halsey; R. J. Levis.

*Obstetricians.*—Drs. R. A. F. Penrose and E. McClellan.

Clinical lectures are delivered every Wednesday and Saturday, by Drs. Agnew and Biddle.

